# Genome-wide discovery and validation of *Eucalyptus* small RNAs reveals variable patterns of conservation and diversity across species of *Myrtaceae*

**DOI:** 10.1186/s12864-015-2322-6

**Published:** 2015-12-29

**Authors:** Marília de Castro Rodrigues Pappas, Georgios Joannis Pappas, Dario Grattapaglia

**Affiliations:** Embrapa Recursos Genéticos e Biotecnologia, Brasília, DF Brazil; Universidade de Brasília, Brasília, DF Brazil; Programa de Pós-Graduação em Ciências Genômicas e Biotecnologia, Universidade Católica de Brasília, Brasília, DF Brazil

**Keywords:** Deep sequencing, *Eucalyptus*, Genome mapping, Interspecific variability, micro RNAs, Myrtaceae, Small RNAs

## Abstract

**Background:**

Micro RNAs are a class of small non coding RNAs of 20–24 nucleotides transcribed as single stranded precursors from MIR gene loci. Initially described as post-transcriptional regulators involved in development, two decades ago, miRNAs have been proven to regulate a wide range of processes in plants such as germination, morphology and responses to biotic and abiotic stress. Despite wide conservation in plants, a number of miRNAs are lineage specific. We describe the first genome wide survey of *Eucalyptus* miRNAs based on high throughput sequencing.

**Results:**

In addition to discovering small RNA sequences, MIR loci were mapped onto the reference genome and interspecific variability investigated. Sequencing was carried out for the two most world widely planted species, *E. grandis* and *E. globulus.* To maximize discovery, *E. grandis* samples were from BRASUZ1, the same tree whose genome provided the reference sequence. Interspecific analysis reinforces the variability in small RNA repertoire even between closely related species. Characterization of *Eucalyptus* small RNA sequences showed 95 orthologous to conserved miRNAs and 193 novel miRNAs. *In silico* target prediction confirmed 163 novel miRNAs and degradome sequencing experimentally confirmed several hundred targets. Experimental evidence based on the exclusive expression of a set of small RNAs across 16 species within Myrtaceae further highlighted variable patterns of conservation and diversity of these regulatory elements.

**Conclusions:**

The description of miRNAs in *Eucalyptus* contributes to scientific knowledge of this vast genre, which is the most widely planted hardwood crop in the tropical and subtropical world, adding another important element to the annotation of *Eucalyptus grandis* reference genome.

**Electronic supplementary material:**

The online version of this article (doi:10.1186/s12864-015-2322-6) contains supplementary material, which is available to authorized users.

## Background

In the last decade, small non-coding RNAs have emerged as key endogenous regulatory elements in eukaryotic cells. It became clear that part of the so called junk DNA was transcribed into silencing RNAs that take part in an intricate gene regulatory network with highly specific functions [1–3]. Among a variety of functional non coding small RNAs (sRNAs), two main classes have been subject of intensive investigation in plants: small interfering RNAs (siRNAs) and micro RNAs (miRNAs). Mature siRNAs are predominantly 24-nt long transcribed by RNA polymerase IV with subsequent double strand synthesis [4, 5]. They are fundamental players in cellular defense mechanisms against viruses [6, 7] and epigenetic regulation, as the drivers of RNA directed DNA methylation (RdDM), contributing to the silencing of transposable elements and transcriptional gene silencing [8, 9]. MiRNAs are 20 to 24-nt long sRNAs involved predominantly in post-transcriptional gene regulation. Single stranded miRNAs primary precursors – pri-miRNAs – are transcribed by RNA polymerase II from MIR genes [4], and processed by Dicer like 1 enzyme (DCL1) to generate an intermediary precursor (pre-miRNA), typically folded into a stable single strand stem loop structure [1, 10–12]. In plants, the mature miRNA is excised from the pre-miRNA, exported from the nucleus and then incorporated into protein RNA induced silencing complex (RISC). RISC is guided to the target messenger RNA (mRNA) by sequence complementarity to miRNA [13, 14]. The silencing mechanism is determined by the degree of complementarity between the pair miRNA-target mRNA. Pairing mismatches are frequent in metazoans and miRNAs tend to block target mRNA by hindering its translation whereas high complementarity usually directs mRNA degradation in plants [15]. A wide variety of processes such as plant development [16–19], flowering [18, 20, 21], meristem and vascular differentiation [16], disease resistance [22–24] and response to abiotic stress [25–27] are regulated by miRNAs.

One of the first steps to ascertain how these components engage in complex regulatory networks is the characterization of the small RNA repertoire of a species. In recent years, second generation sequencing provided the technical breakthrough for rapid and comprehensive small RNA discovery including non-conserved and low abundance miRNAs [18, 26–36]. Accordingly, a surge in studies characterizing plant sRNAs was initiated. Despite the vast number of sRNA reads in sequencing data, *bona fide* plant miRNA genes of a given species are typically numbered in hundreds exemplars. Then extensive post-processing becomes fundamental to adhere sRNA sequences to a strict set of rules defining miRNA genes, which have foundations in recalling their biogenesis mechanisms [37].

There are many examples of highly conserved miRNAs families in plants, some present from basal plant species to angiosperms [38]. However it is known that there is great interspecific variability in small RNAs repertoire with several MIR genes lineage or species specific. Studies on miRNA discovery, either by RNA sequencing or *in silico* prediction, have shown this high diversity in different species [39–42].

Among plant miRNAs present in the database miRBase [43], forest and fruit trees still have very few representatives aside from Poplar (*Populus trichocarpa)* [44]. Recent miRNA discovery studies were published for apple [45], *Pinus densata* [46], the rubber tree *Hevea brasiliensis* [47], peach [48], Chinese fir [35], *Carya cathayensis* [18] and *Eugenia uniflora* [34].

*Eucalyptus* is a highly diverse genus of the *Myrtaceae* family. Native to Australia and its northern islands, *Eucalyptus* species occur predominantly in the southern hemisphere from sea level to alpine tree line and from high rainfall to semi-arid zones [49]. *Eucalyptus* species are mostly outcrossers [50, 51] and their extensive genetic variation has been widely used in breeding programs [52]. Due to their noteworthy high growth rate, wide adaptability, high biomass production and carbon sequestration capabilities, eucalypts became the hardwood crop most widely planted in tropical and subtropical areas, exceeding 20 million hectares around the world [53]. *Eucalyptus globulus* and *Eucalyptus grandis* are currently the most extensively planted species among the ~700 species described for the genus, widely used as sustainable short fiber source for pulpwood and energy. Despite the great wood quality of *E. globulus* for pulp production, its temperate origin implies poor adaptation to highly productive tropical environments where *E. grandis* is the species of choice, making this latter one the most widely planted *Eucalyptus* species.

The genome of *E. grandis* was recently sequenced [54] and is available at Phytozome [55]. As part of that landmark development we performed a comprehensive annotation of miRNA genes, now fully described in this report. This additional layer of information will be valuable to promote a deeper genomic understanding of a number of processes such as tree growth, vascular development, phase change and response to environmental stresses, pests and pathogen, where miRNAs are known to be involved, and that currently make up the bulk of investigation both in forest productivity and health. Complementary strategies were used, starting with Illumina-based high throughput small RNA library sequencing (smRNA-Seq) for *E. grandis* and *E. globulus,* followed by degradome sequencing analyses for large-scale target mRNA identification. This study represents the first genome wide discovery, mapping and characterization of *Eucalyptus* miRNAs and should provide useful fundamental information for upcoming studies on gene regulation in what has now been promoted to an additional model species for forest tree genomics.

## Results

### Small RNA sequencing data

Illumina GAII deep sequencing was carried out for the small RNA fraction of four samples: one leaf (*E. grandis* BRASUZ1) and three developing xylem samples (*E. grandis* BRASUZ1 and two *E. globulus* trees). This experiment resulted in a total of 6,104,498 raw reads ranging from 1,115,404 to 1,766,355 per sample. Pre-processing steps, namely quality screening, adapter and redundancy removal, resulted in a total of 1,857,986 unique sequences (Table 1). Contaminant sequences of ribosomal, chloroplast and tRNA origin were filtered out totaling 1.8 % of the unique sequences.

**Table 1:** Overall sequencing counts of small RNA sequecing in Illumina GAII platform

Sample	Total raw reads	Filtered reads	Unique sequences
*E. grandis* BR1 - leaf	1.484.867	1.450.215	265.579
*E. grandis* BR1 - xylem	1.766.355	1.695.542	377.958
*E. globulus* A2	1.737.872	1.703.806	377.958
*E. grandis* C3	1.115.404	1.093.630	476.080
Total	6.104.498	5.943.193	1.874.515

Filtered reads refer to number of reads after filtering for low quality and no adapter sequences

**Fig. 1:**
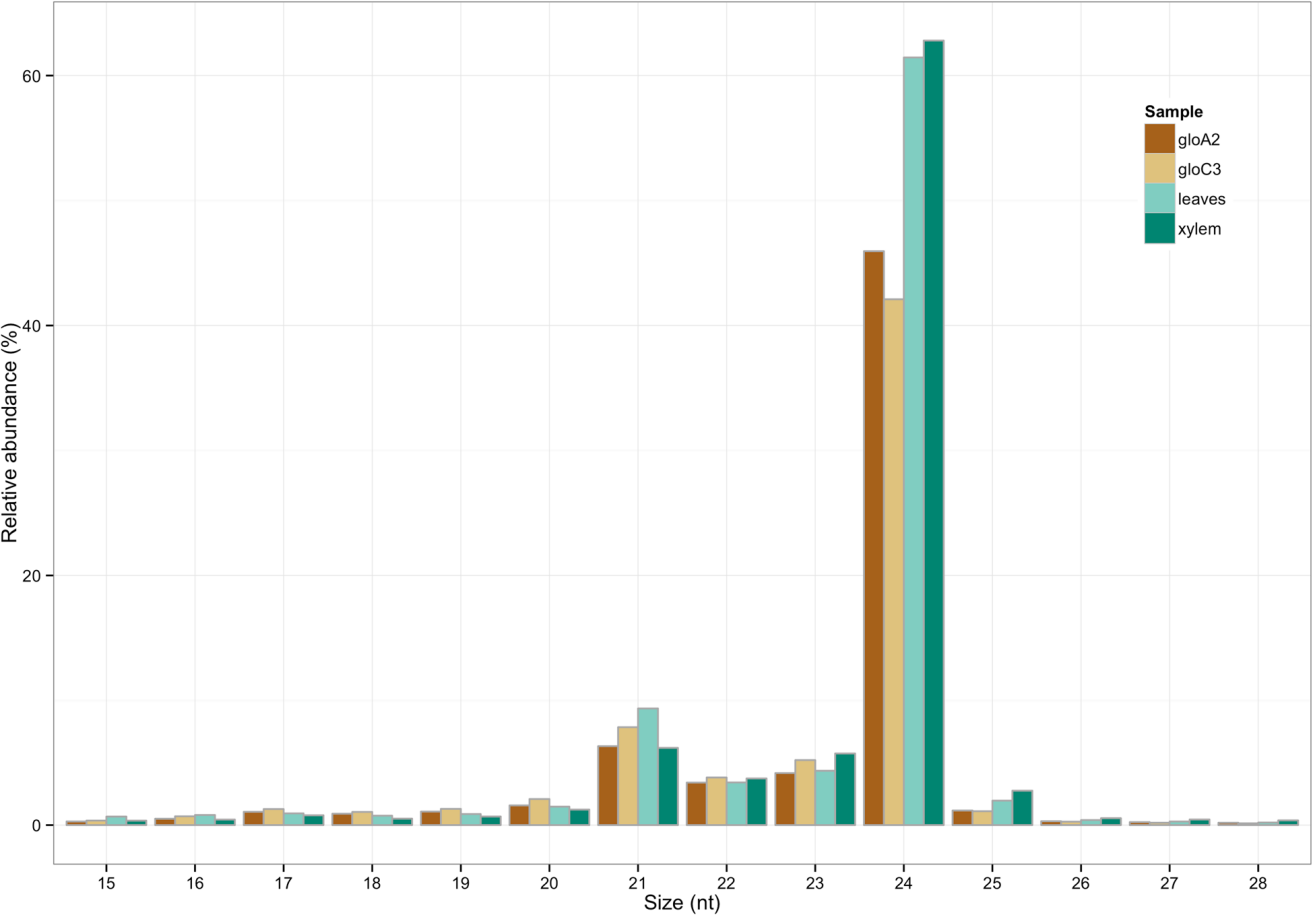
Distribution of smRNA-Seq reads by sequence length. Comparative read size (in nucleotides –nt) distribution abundance for all four samples in smRNA-Seq: *E. globulus* A2 developing xylem (gloA2), *E. globulus* C3 developing xylem (gloC3), BRASUZ BR1 leaves (leaves) and BRASUZ BR1 developing xylem (xylem)

**Fig. 2:**
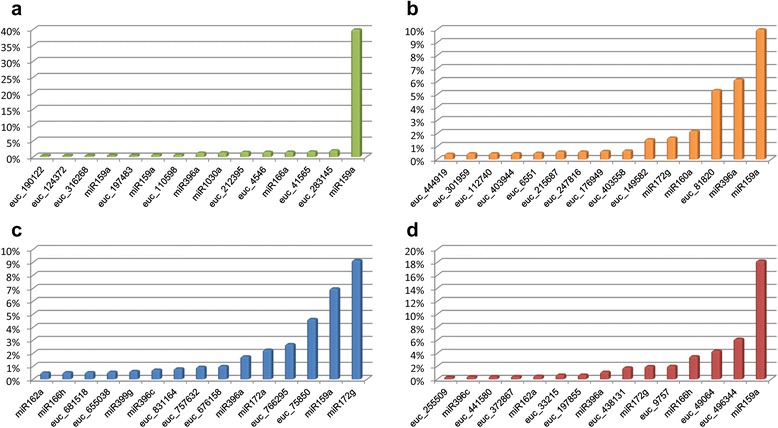
Most abundant 21 nucleotide (21-nt) reads in smRNA-Seq. Frequency histograms showing percentage distribution of 15 most abundant 21-nt small RNA reads per sample: BRASUZ BR1 leaves (**a**), BRASUZ BR1 developing xylem (**b**), *E. globulus* A2 developing xylem (**c**) and *E. globulus* C3 xylem (**d**). Small RNA sequences are named as conserved miRNAs or *Eucalyptus* specific sRNAs (euc)

Read size distribution shows the expected two main peaks at 21 and 24 nucleotides (nt). Twenty-four nucleotide sequences are the most abundant reads in all four samples (Fig. 1). As seen by size ranking of smRNA-Seq data, 24-nt sequences also show extensive sequence diversity with the highest number of unique sequences (clusters) (Additional file 1: Figure S1). Despite the high diversity, each 24-nt cluster exhibits low expression level – none of the 24-nt read makes up 1 % of the total counts in the size class. Twenty-one nucleotide sequences show an opposite distribution showing less sequence diversity (fewer unique sequences) but the highest counts per cluster observed amongst all sRNA size classes analyzed (from 15 to 28-nt). The most abundant sequence within the 21-nt class varies from 10 % up to 40 % of total counts in BRASUZ1 leaves sample (Fig. 2).

**Fig. 3:**
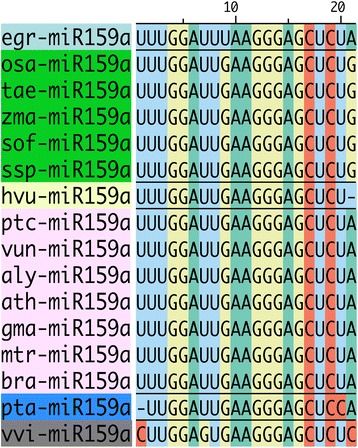
Conservation of miR159a. Multiple sequence alignment of *Eucalyptus grandis* miR159a (egr-miR159a) and plant miR159a sequences from miRBase (sequence id as in miRBase)

Conserved miRNAs were often observed among 21-nt reads with highest counts. MiR159a was consistently the most abundant 21-nt sequence in three out of four samples – *E. grandis* BRASUZ1 leaves (40 %) (Fig. 2a) and developing xylem (10.5 %) (Fig. 2b), and *E. globulus* C3 developing xylem (18.2 %) (Fig. 2d). In the *E. globulus* A2 xylem sample, miR159a (6.99 %) was outnumbered by miR172g (9.16 %), but was the second most abundant 21-nt read (Fig. 2c). MiR159a is a highly conserved miRNA as seen by alignment of *Eucalyptus* miR159a with all plant orthologs present in miRBase (Fig. 3). MiR166 and miR396 are other conserved miRNAs that are featured among the top fifteen 21-nt reads in all four samples. A broad search for conserved miRNAs was carried out and is discussed further.

### Genome mapping

Mapping of 1,857,986 sRNA unique sequences on the *E. grandis* reference genome (Phytozome 8.0) was carried out using BWA [56]. Uniquely mapped reads per sample varied from 67.2 % to 87.3 % (Table 2). Mapping data confirmed the high diversity of the 24-nt sRNA sequences previously mentioned in total reads counting. For all samples, 24-nt reads represent the highest number of uniquely mapped sequences.

**Table 2:** Mapping data of unique sequences from smRNA-Seq to the *Eucalyptus grandis* genome using BWA

Sample	BR1 leaves	BR1 xylem	*E. globulus* A2	*E. globulus* gloC3
Mapped sequences	231.893 (87,3 %)	327.580 (86,7 %)	507.430 (67,2 %)	321.403 (67,5 %)

**Fig. 4:**
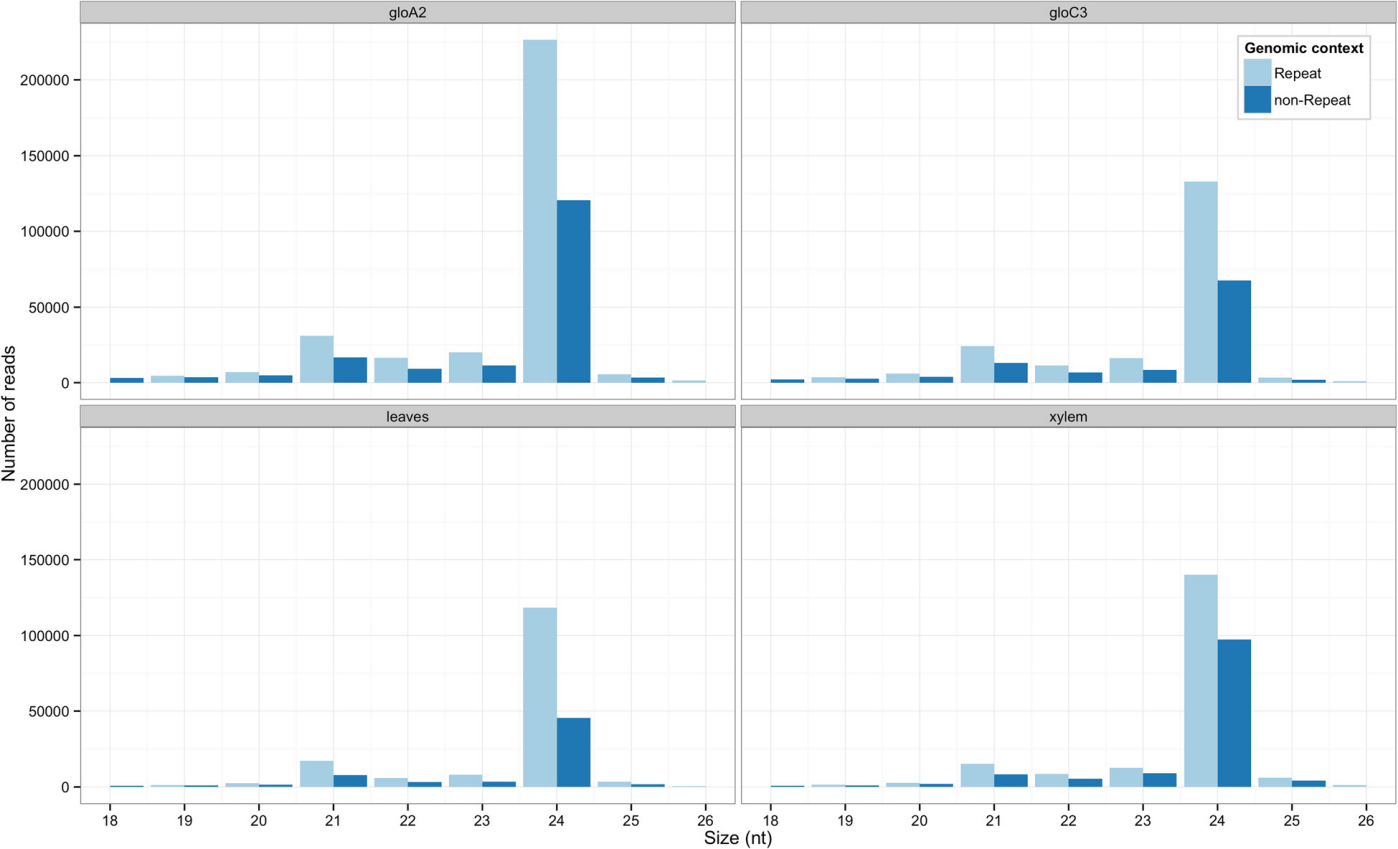
Mapping of small RNA reads to repetitive regions of *Eucalyptus grandis* reference genome. Size distribution (in nucleotides – nt) of small RNA reads from smRNA-Seq data based on mapping to repetitive regions of *Eucalyptus grandis* reference genome per sample: *E. globulus* A2 developing xylem (gloA2), *E. globulus* C3 developing xylem (gloC3), BRASUZ BR1 leaves (leaves) and BRASUZ BR1 developing xylem (xylem). Light blue bars represent reads mapped to repetitive regions and dark blue bars, to non-repetitive regions

Mapping data was investigated for correlation between sequence size and mapping location in repetitive regions. Mapping data of unique reads from 19 to 26-nt showed sRNA reads located mostly in repeat regions (70.1 % in *E. grandis* BRASUZ1 leaves, 59.3 % in *E. grandis* BRASUZ1 xylem, 64.3 % in *E. globulus* A2 xylem and 64.9 % in *E. globulus* C3 xylem). Size distribution of mapped reads revealed a general tendency to mapping on repetitive regions irrespective of the sequence size (Fig. 4).

### Characterization of conserved miRNA sequences

A similarity search of all 20 to 22 nucleotides unique sequences was done using PatMan [57] against miRBase plant mature miRNA sequences. A total of 303 distinct 21-nt sRNAs showed significant similarity (with at most three mismatches) to an orthologous miRNA sequence in miRBase. Conserved reads with 100 % identity totaled 95 sequences encompassing 25 miRNA families (Additional file 2: Table S1). Expression of miRNAs miR156a, miR159a, miR160 and miR172b was assessed by northern blot in leaves and developing xylem of *E. dunnii*, *E. urophylla* and *E. grandis* BRASUZ1 (Additional file 3: Figure S2).

MiRNAs with 22-nt in length constitute a class of sRNAs not as abundant as the 21-nt ones but which make up a subclass with outstanding role in triggering biosynthesis of phased secondary siRNAs known as tasiRNAs (trans-acting siRNAs) or phasiRNAs (phased siRNAs) [58, 59]. Sixteen 22-nt conserved miRNA sequences with 100 % identity and 69 with up to three mismatches were identified.

### *In silico* prediction by miRDeep

There are hundreds of MIR genes identified in plant genomes. The massive bulk of smRNA-Seq reads thus requires careful analysis to identify *bona fide* miRNA genes, as established by a set of strict criteria [37]. The first one is to be excised from a stem loop arm of a single stranded intermediary precursor (pre-miRNA)*.* To test smRNA-Seq data for this criterion, the reference genome of *E. grandis* BRASUZ1 provided the ideal conditions for a broad computational search. We used miRDeep2 package for *de novo* prediction of miRNAs from sequencing data [60]. After a genome-wide search for candidate regions complying with precursor secondary structure constraints, 193 mapped sequences showed to be compatible with a MIR gene locus (Additional file 4: Table S2). Eighty two of these in *E. grandis* BRASUZ1 leaves sample, 55 in *E. grandis* BRASUZ1 xylem, 74 and 73 in *E. globulus* A2 e C3 xylem samples, respectively.

Five of the most abundant 21-nt reads in smRNA-Seq data (euc_sRNA_149582, euc_sRNA_75850, euc_sRNA_438131, euc_sRNA_33215 and euc_sRNA_372867) (Fig. 2) had their expression experimentally revalidated by northern blot in three *Eucalyptus* species – *E. dunni*, *E. urophylla* and *E. grandis* (Additional file 5: Figure S3). Nevertheless, despite the evidences of expression (smRNA-Seq and blot) and mapping on the reference genome of *E. grandis*, their flanking regions did not meet the requirements for a typical miRNA precursor structure.

### Target prediction and degradome sequencing

**Fig. 5:**
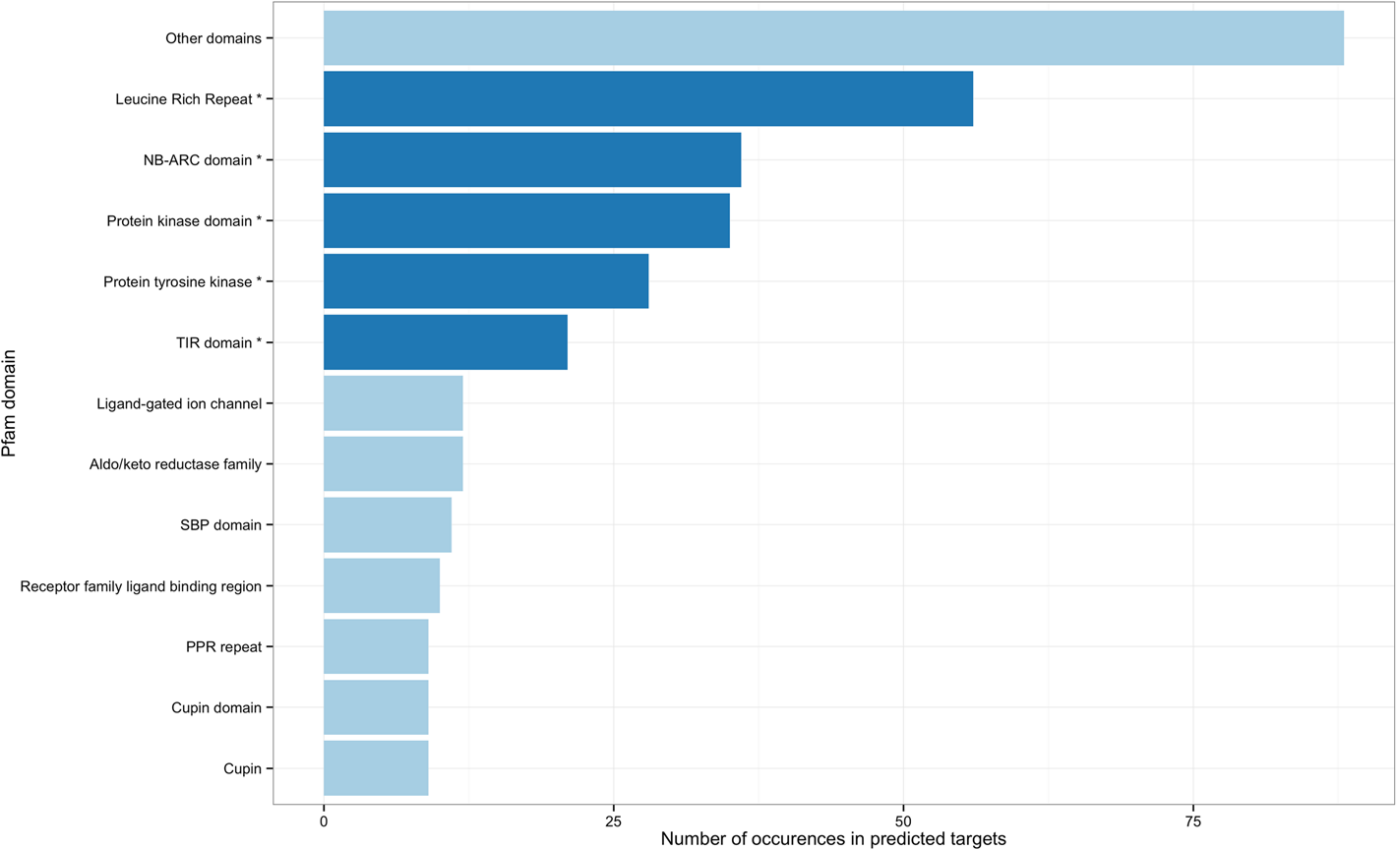
Enrichment of protein domains in predicted micro RNA targets. Count of protein domains in messenger RNA targets predicted by psRNATarget. Protein domains typical from disease resistance genes family TIR-NB-LRR (_*_) are the most abundant

Prediction of transcripts targeted by miRNAs adds another level of *in silico* confirmation, providing clues about potential biological processes being regulated. Target prediction was performed for all mapped sequence candidates with a compatible precursor secondary structure using psRNATarget [61]. The *Eucalyptus* transcript database from Phytozome was used for reverse complementary matching between smRNA-Seq data and potential target transcripts. Functional annotation of targets was retrieved from the BioMart tool available in Phytozome. The number of predicted targets for each miRNA candidate varied from one to twenty transcripts. The enrichment for specific protein domains in predicted target mRNAs is shown in Fig. 5 and indicates the prevalence of signature domains for NB-LRR (NB-ARC, leucine rich repeat) disease resistance genes, ion transporters, SBP (squamosa binding proteins) transcription factors and PPR (pentatricopeptide repeat) proteins. Predicted targets also include transcripts related to wood formation such as cellulose synthases and cytochrome P450 which is involved both in biosynthetic and detoxification pathways.

**Fig. 6:**
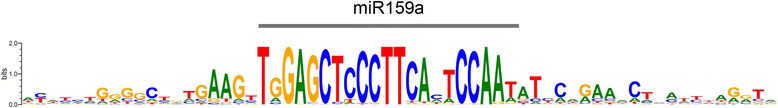
Sequence logo of MYB transcription factor transcripts retrieved from Phytozome 10.3 highlighting miR159a binding site (created with WebLogo 3). Each transcript is shown with 20 nucleotides flanking the binding site

Target prediction for miR159a, the most abundant 21-nt read in our smRNA-Seq data, resulted in 10 predicted transcripts (Additional file 6: Table S3) including two MYB transcription factors (Eucgr.G03183.1 and Eucgr.E01581.1). To get a glimpse of miRNA binding site conservation on MYB transcription factors, the orthologs were searched in Phytozome database, the transcripts scanned for the presence of miR159a binding site, using psRNATarget, and the ones with at least 20 bases flanking the binding the site were used to create a sequence logo (using WebLogo 3 [62]) highlighting the miRNA binding site at the target transcripts (Fig. 6).

To experimentally confirm *in silico* predicted targets, a degradome sequencing experiment was performed. Libraries of 3’ cleavage products were prepared from mRNA samples of leaf and developing xylem from an adult *E. grandis* BRASUZ1 tree. Illumina HiSeq sequencing of both samples resulted in 27,244,395 raw reads. After filtering for low quality reads, including no adapter, no insert and sequences smaller than 18 nucleotides, the total number was reduced to 26,387,851 (97.53 %) valid sequence reads. Annotation of filtered reads resulted in 18,257,616 total and 2,887,536 unique sequences. Non coding RNAs – such as ribosomal, transporter, small nuclear and small nucleolar – accounted for around 2.67 % of the total read count. Sharing of unique sequences between the samples represented 9.64 % of the reads.

Analysis of degradome sequencing data with CleaveLand pipeline identified 189 cleaved transcripts targeted by 21-nt sRNA sequences from leaves and 324 from xylem smRNA-Seq data (Additional file 7: Table S4; Additional file 8: Table S5). Considering the 22-nt sRNAs, the number of pairs was 149 and 248, respectively. Targets included a set of transcription factors such as MYB, GRAS and SBP families, cation transporter genes and ARFs (auxin response factors) as reported before for other plant species [63, 64] and matching the *in silico* results predicted by psRNATarget.

A wide variety of other transcripts involved in diverse physiological processes such as cytochrome P450, TIR-NB-ARC disease resistance genes and nuclear transport factor 2 (NTF2) were also identified in the degradome data. Some results corroborate known targets of conserved miRNAs such as a SBP transcription factor cleaved by miR156 [65–67], an ARF transcript, by miR160 [68, 69] and the transcription factor AP2 (apetala 2), by miR172 [70, 71]. The degradome sequencing data further supported two newly identified *Eucalyptus* miRNAs, which displayed two distinct transcripts of cellulose synthase as targets, detected only in the xylem sample (Eucgr.D00476.1 and Eucgr.H00939.1 in Phytozome).

### Conservation within *Myrtaceae*

Interspecific variability in sRNA loci was observed from mapping data of *E. grandis* and *E. globulus* sRNA reads on the *E. grandis* reference genome. In our smRNA-Seq data, the number of mapped sequences differed by 20 % between the two species (Table 2). In order to extend the inquiry of sRNA conservation within Myrtaceae, we expanded the analysis to other species and genera. A comparative analysis of *Eucalyptus* and *Eugenia uniflora* smRNA-Seq data (available at the NCBI Gene Expression Omnibus, GEO, accession number GSE38212) was carried out. Mapping of sRNA reads of *E. uniflora* to the *E. grandis* genome totaled 1,392,334 (34.7 %) of the total unique sequences. Size sorting of mapped sequences showed a higher conservation of 21-nt sRNAs and lower of 24-nt, 61.6 % and 21.1 % respectively (Table 3). Same tendency was observed when overall unique sequences were compared. Considering only perfect matches (100 % identity), 21-nt common sequences were 10 fold higher than 24-nt and allowing 1 mismatch, 7 fold higher. Higher conservation was also observed for 22 nucleotides sRNAs when compared to 24 nucleotides.

**Table 3:** Comparative analysis of *Eucalytpus* and *Eugenia uniflora* small RNA sequencing data

Sequence lenght (nt)	*Eugenia* and *Eucalyptus* overall common sequences	*Eugenia* sequences mapped to E*. grandis*	*Eugenia* unique sequences
21	7,018 (1.2 %)	352,691 (61.6 %)	572,311
22	4,742 (1.7 %)	121,025 (42.9 %)	282,335
24	5,231 (0.2 %)	514,683 (21.1 %)	2,436,699
Total	16,991 (0.5 %)	988,399 (30.0 %)	3,291,345

Number of unique sequences common to both *E. uniflora* and *Eucalyptus* (no mismatches), number of mapped unique sequences of *E. uniflora* small RNAs on the *E. grandis* genome and total number of *E. uniflora* unique sequences

**Fig. 7:**
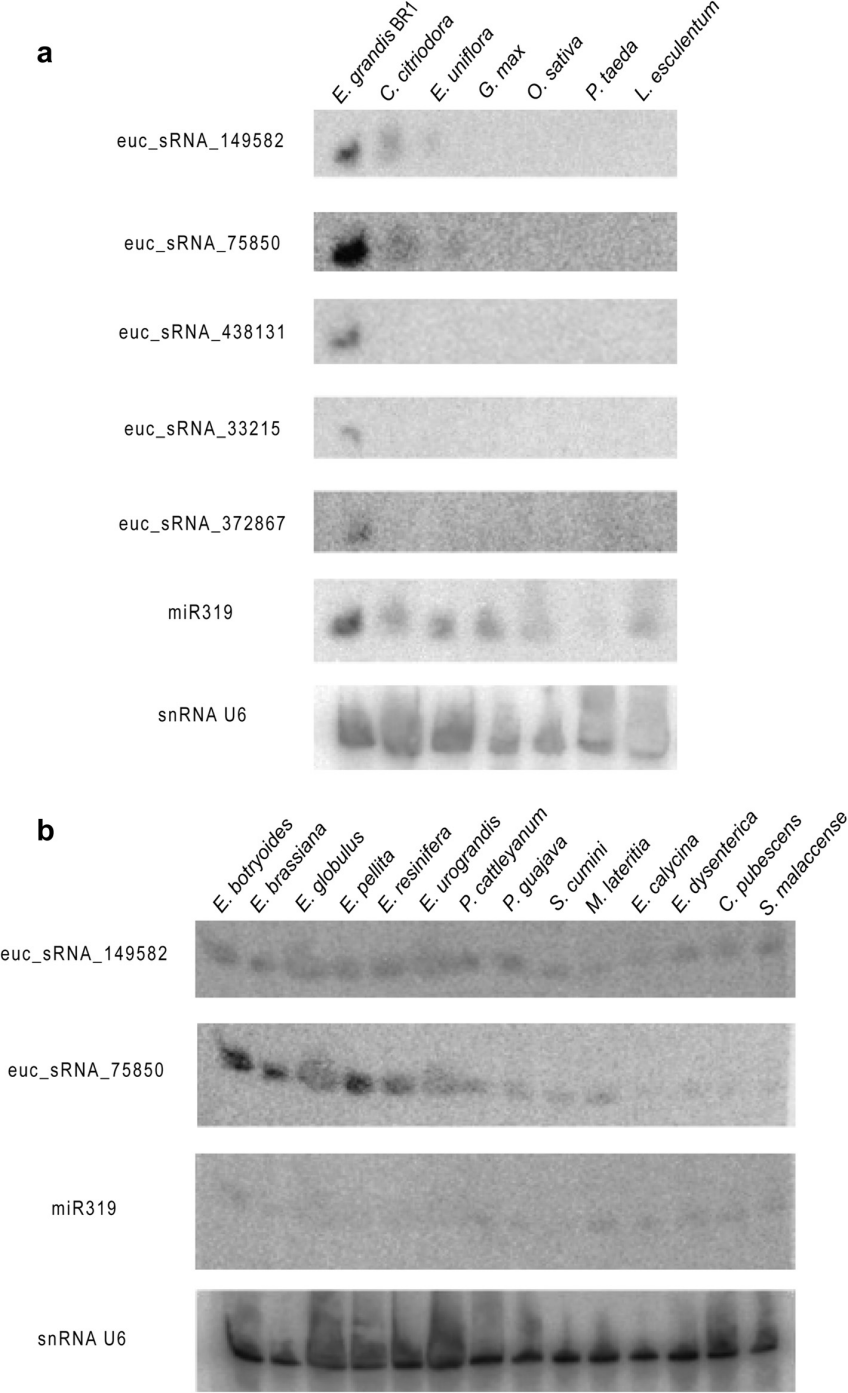
Conservation of small RNAs within Myrtaceae family species and unrelated outgroups via expression validation. Northern blot analysis of some of the most abundant 21-nt sRNAs in smRNA-Seq was assessed in leaves of *Eucalyptus grandis* BRASUZ1 (*E. grandis* BR1), *Corymbia citriodora*, *Eugenia uniflora*, *Glycine max*, *Oryza sativa*, *Pinus taeda* and Lycopersicum esculentum (**a**); *Eucalyptus botryoides*, *E. brassiana*, *E. globulus*, *E. pellita*, *E. resinifera*, *E. urograndis* (hybrid of *E. urophylla* x *E. grandis*), *Psidium cattleyanum*, *Psidium guajava*, *Syzygium cumini*, *Melaleuca lateritia*, *Eugenia calycina*, *Eugenia dysenterica*, *Campomanesia pubescens* and *Syzygium malaccense* (**b**). Micro RNA miR319 was blotted as positive control and snRNA U6 as loading control

Northern blot analysis of some of the most expressed sRNA reads in the smRNA-Seq data was carried out in order to investigate conservation within the *Myrtaceae* family. Five sRNAs probes were hybridized against RNA from completely developed leaves of seventeen *Myrtaceae* species, including six *Eucalyptus* species (*E. grandis*, *E. botryoides*, *E. brassiana*, *E. globulus*, *E. pellita* and *E. resinifera*), a hybrid *E. urograndis* (*E. urophylla* x *E. grandis*), in addition to ten other species of different genera of *Myrtaceae* – *Corymbia citriodora* (previously classified as *Eucalyptus citriodora*), *Eugenia uniflora*, *Psidium cattleyanum*., *Psidium guajava*, *Syzygium cumini*, *Melaleuca lateritia*, *Eugenia calycina*, *Eugenia dysenterica*, *Campomanesia pubescens* and *Syzygium malaccense*. Three diverse selected outgroup species – *Glycine max*, *Lycopersicum esculentum* and the gymnosperm *Pinus taeda* – were also included in the experiment. Expression of two sRNAs (euc_sRNA_149582, euc_sRNA_75850) were not detected in any of the outgroup species (Fig. 7a) but were consistently detected in all *Myrtaceae* samples (Fig. 7b). The other three sRNAs (euc_sRNA_438131, euc_sRNA_33215 and euc_sRNA_372867) are potentially *Eucalyptus* specific as their expression were confirmed in three *Eucalyptus* species – *E. grandis*, *E. dunnii* and *E. urophylla* – (Additional file 5: Figure S3) but not in the outgroups *G. max*, *O. sativa*, *L. esculentum* and *P. taeda* nor in the other *Myrtaceae* species tested – *C. citriodora* and *E. uniflora* (Fig. 7a). Secondary structure prediction for genome flanking regions of these sRNAs failed to confirm them as miRNAs.

## Discussion

Our results highlight the pivotal importance of a careful analysis and raw data filtration of the massive amounts of sequence data produced by next generation sequencing. The large initial number of reads is progressively reduced after consecutive analyses. Ultimately, 1,405,134 (75.63 % of total valid reads) 21-nt unique sequences mapped unambiguously to the *E. grandis* BRASUZ1 reference genome. Ninety five 21-nt unique sequences are conserved micro RNAs with 100 % identity to sequences available in miRBase. Three hundred and three have an orthologous sequence in miRBase with up to 3 mismatches and thus potentially correspond to new isoforms of conserved miRNA families. While the identification of conserved miRNAs can be easily accomplished by similarity search with previously described orthologous sequences, the annotation of new miRNAs requires the adherence to a set of strict criteria such as the presence of a compatible secondary structure of precursor sequence and target prediction. This process was highly benefited by the availability of a high quality *E. grandis* reference genome, possibly the last one generated exclusively by Sanger sequencing. Among all 21-nt mapped sequences, 193 have a compatible miRNA precursor secondary structure as predicted by miRDeep2. From this subset, 163 21-nt sequences had at least one predicted target by psRNATarget, complying with the criteria to annotate a miRNA.

As seen by the read size distribution, 24-nt reads were the most abundant size class in the smRNA-Seq data with up to 3.75 times the number of 21-nt reads. The predominance of 24-nt sRNAs, mainly represented by siRNAs, is well known in angiosperms and had been previously reported for several species [4, 72–74]. Noteworthy is that, in addition to being the most abundant class overall, 24-nt sRNA *Eucalyptus* sequences constitutes by far the most diverse group, with the greatest number of clusters. The 24-nt overall high sequencing read counts observed is therefore explained by its diversity of unique sequences even with few reads per cluster when compared to 21-nt reads. Altogether this is in agreement with the premise that small RNA repertoire in plants is dominated by a vast number of 24-nt small interfering RNAs (siRNAs) [15]. Contrary to that, 21-nt reads – which include both siRNAs and miRNAs – are less diverse, with fewer unique sequences, but exhibit the highest read counts per cluster in sequencing data.

As heterochromatic 24-nt sRNAs predominantly silence transposons and repeat regions by directing DNA methylation at complementary sites in the genome [15], one would expect to see a higher proportion of these sRNAs mapping to repetitive regions. In fact this seemed to be an overall tendency for all *Eucalyptus* sRNAs from 19 to 26-nt as observed for all samples in the smRNA-Seq.

Interspecific variability in small RNA content of the genome was evidenced by the mapping data. *E. grandis* BRASUZ1 displayed 20 % higher proportion of uniquely mapped sequences to its own genome when compared to *E. globulus*. Pairwise comparison of smRNA-Seq data for all samples corroborated this assertion: the most similar samples were from developing xylem of different trees of *E. globulus*. Pairwise comparison also highlighted the tissue specificity of sRNA expression, as the most diverse repertoire of sRNA reads were from distinct tissues– developing xylem and leaves – collected simultaneously from the same *E. grandis* BRASUZ1 tree (data not shown).

It is suggested that conserved miRNAs usually have higher expression and that lineage specific ones are expressed at lower levels or in specific tissues, developmental stages or conditions [29, 75]. Counting of our 21-nt sequences reinforced this concept, as conserved miRNAs were consistently seen among the most abundant reads. Though a large number of 21-nt sRNAs that are not conserved miRNAs were also highly expressed, *in silico* analysis of these sequences showed that they do not fit canonical miRNA criteria being representatives of another class of sRNAs.

Recently, a large scale identification of miRNAs was performed in *Eugenia uniflora* [34]. A comparative analysis between smRNA-Seq data from *Eucalyptus* and *E. uniflora* indicated a high conservation within Myrtaceae family. This high conservation probably arises mostly from conserved miRNAs sequences as evidenced by higher conservation of 21-nt sequences. MiRNAs commonly have non-related targets and are frequently involved in housekeeping conserved pathways. As miRNA silencing relies on sequence complementarity to heterologous targets, this class tends to suffer more selective evolutionary pressure. On the other hand, siRNAs usually silence related targets or even their own origin loci, acting in cis. It is suggested that siRNAs suffer little or no selective pressure to maintain sequence conservation resulting in high evolutionary rates [76]. Our experimental results of northern blot for 21-nt sRNAs (other than miRNAs) assessed in various Myrtaceae species and outgroups suggested a tendency of sequence conservation of highly expressed sRNAs within the family.

Target prediction showed predominance of NB-LRR proteins, the most common disease resistance genes in plants, known to be highly regulated by sRNAs [77]. Transcription factor families, as SBP and MYB, were also abundantly present in target prediction. MYB proteins are known as transcription factors related to wood formation [78]. The R2R3-MYB gene family, as an example, is supposed to control lignification during xylogenesis (wood growth) [79, 80]. Targets involved in biosynthetic pathways were also predicted such as cytochrome P450. These proteins play a key role in the synthesis of structural polymers as lignins [81] which together with cellulose are the two basic wood components.

A recent study on *Eucalyptus grandis* miRNAs investigated the relationship between alterations in miR156 and miR172 expression, associated with vegetative phase change [19, 82, 83], and adventitious root induction during development [84]. Inquiries like this highlight the potential of miRNA investigation in diverse biological pathways with a vast impact on plant development and productivity among other aspects. That study also conducted a profile on miRNA expression in cuttings describing 40 known and 8 novel miRNAs, including one (Cluster_41475) also present in our data (Scaffold11 - 40625339–40625414).

## Conclusions

This work provides the first comprehensive genome-wide discovery and characterization effort of miRNA in species of *Eucalyptus*. High throughput smRNA-Seq with *in silico* and experimental evidences allowed the characterization of conserved and novel miRNAs. Due to the lack of biological replicates in our smRNA-Sequencing data, an addition that would have allowed further quantitative analyses, we limited our study to a qualitative survey of miRNAs. Nevertheless, the data presented lays the foundation for forthcoming differential miRNA expression analyses.

The availability of a high quality genome sequence for *E. grandis* was a key asset to carry out a robust precursor structure prediction and this in turn provided improved experimental evidence to support the discovery of bona fide novel miRNAs. When a reference genome is not available, precursor secondary structure prediction is dependent on the availability of expressed sequence tags (ESTs) a method that has limitations. Furthermore, by using smRNA-Seq obtained from the same exact tree whose genome is the reference genome sequence, the analysis considerably improved the number of mapped sequences. The smRNA-Seq data from *E. globulus* on the other hand, provided solid evidence confirming the interspecific variability in the small RNA repertoire even between related species belonging to the same subgenus Symphyomyrtus. Availability of a large transcript database of the target species also highly favored the identification and characterization of targets. The identification, mapping and characterization of miRNAs loci described in this work directly contributed to the annotation of the *Eucalyptus grandis* genome [54] adding another layer of information to the current reference genome.

## Methods

### Plant material, RNA extraction and sequencing

Plant material for smRNA-Seq includes the same tree genotype used by JGI-DOE (Joint Genome Institute – USA Department of Energy) for whole genome sequencing: *E. grandis* (BRASUZ1 tree), a selfed tree (S_1_) from Suzano Group (Brazil). RNA from four biological samples was prepared for deep sequencing experiments: developing xylem and leaves of *E. grandis* BRASUZ1 and developing xylem of two unrelated *E. globulus* trees (named A2 and C3). RNA extraction was performed with an adapted CTAB protocol [85]. Library preparation for Illumina GAII sequencing used single end kit and barcoded adapters to multiplex samples in one lane run, all performed by Fasteris SA (Switzerland). For experimental validation via northern blot, developing xylem and leaves from two trees of *E. dunnii* and two of *E. urophylla* and leaves from two clones of *E. grandis* BRASUZ1 were employed. All RNA samples were obtained from fully developed leaves from adult trees.

### Analytical pipeline

A custom-made computational pipeline was developed to process sRNA sequencing data. A pre-processing step cleans the sequences by trimming sequencing adaptors, quality screening and contaminant removal (including ribosomal, chloroplast and tRNA). Processed reads were size sorted, quantified (tag counting) and used to create a non-redundant read set using UCLUST [86]. The non-redundant reads were mapped to the *E. grandis* reference genome available in Phytozome [55] using BWA [56], with parameters –n 1. In order to identify conserved miRNA sequences, a similarity search using PatMan [57] was carried out against the subset of plant-specific mature sequences from miRBase release 19 [43, 87], allowing at most three mismatches (paramenters –e 3). Novel miRNAs were predicted using the miRDeep2 pipeline [60] (mapper parameters –e –j –l 19 –o 16), which performs a genome wide search for potential miRNA precursors based on the extension of genome regions around mapped reads followed by secondary structure prediction and stability evaluation. Messenger RNA target prediction was performed using psRNATarget [61] applying default settings against the *E. grandis* transcript database from Phytozome server (version 8.0) [55].

### Degradome sequencing

Total RNA from *E. grandis* BRASUZ1 developing xylem and leaves was used for parallel analysis of RNA ends (PARE) library preparation followed by Illumina HiSeq sequencing (BGI – Hong Kong). Analysis of degradome sequencing was performed by CleaveLand 3 pipeline that outputs potentially cleaved sRNA targets from both degradome and smRNA-Seq data [88].

## Additional files

Additional file 1: Figure S1. Size rank profile of smRNA-Seq data. Small RNA sequence clusters illustrating the number of clusters per sequence size (nt) and the abundance of reads in each cluster (dots). BRASUZ 1 leaves (leaves), BRASUZ 1 developing xylem (xylem), *E. globulus* A2 developing xylem (gloA2) and *E. globulus* C3 xylem (gloC3). (TIFF 756 kb) Additional file 2: Table S1. Annotation of *Eucalyptus* conserved miRNAs by similarity search against miRBase. (XLSX 33 kb) Additional file 3: Figure S2. Northern blot analysis of conserved micro RNAs. (TIF 4319 kb) Additional file 4: Table S2.
*Eucalyptus* miRNA loci predicted by miRDeep2. (XLS 26 kb) Additional file 5: Figure S3. Assessing small RNA conservation in *Eucalyptus*. (TIF 4142 kb) Additional file 6: Table S3.
*Eucalyptus* miR159a targets predicted by psRNATarget. (DOCX 12 kb) Additional file 7: Table S4. Targets from degradome sequencing for BRASUZ1 21 bp RNAs - leaves. (XLS 30 kb) Additional file 8: Targets from degradome sequencing for BRASUZ1 21 bp RNAs - xylem. (XLS 50 kb)

## Abbreviations

AP2: Apetala 2
ARFs: Auxin response factors
ESTs: Expressed sequence tags
GEO: Gene Expression Omnibus
miRNA: micro RNA
mRNA: Messenger RNA
NB-LRR: NB-ARC, leucine rich repeat
nt: Nucleotides
NTF2: Nuclear transport factor 2
phasiRNA: Phased siRNA
PPR: Pentatricopeptide repeat
pre-miRNA: Intermediary miRNA precursor
pri-miRNA: Primary miRNA precursor
RdDM: RNA directed DNA methylation
RISC: RNA induced silencing complex
SBP: Squamosa binding proteins
siRNA: Small interferent RNA
smRNA-Seq: Small RNA library sequencing
sRNA: Small RNA
tasiRNA: Trans-acting siRNA
