# Supplementary figures and images for: Genome-wide discovery and validation of Eucalyptus small RNAs reveals variable patterns of conservation and diversity across species of Myrtaceae
Source: BMC Genomics. 2015 Dec 29;16:1113. doi: 10.1186/s12864-015-2322-6 (PMC4696225; doi:10.1186/s12864-015-2322-6)

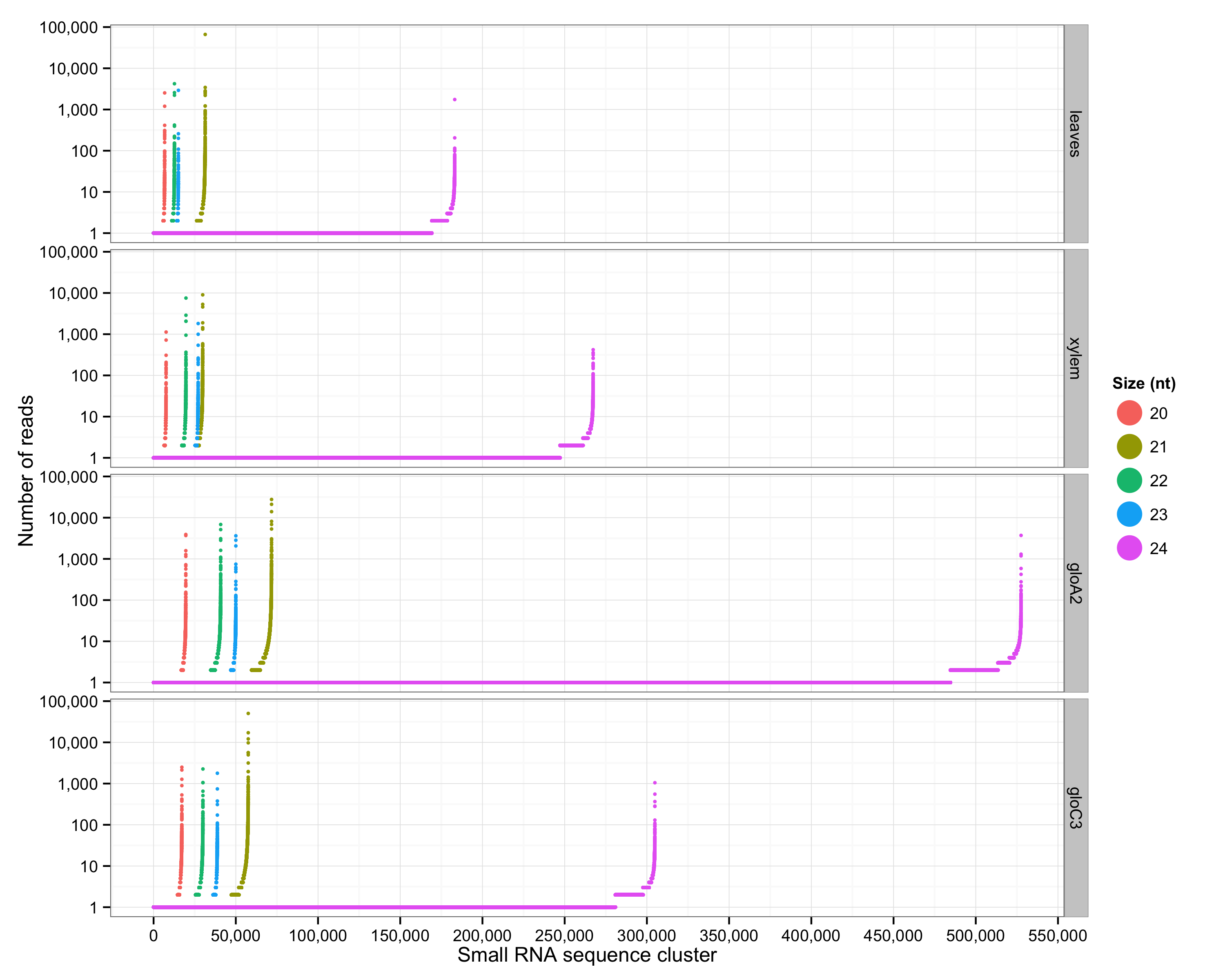

Supplement: Additional file 1: Figure S1. — Size rank profile of smRNA-Seq data. Small RNA sequence clusters illustrating the number of clusters per sequence size (nt) and the abundance of reads in each cluster (dots). BRASUZ 1 leaves (leaves), BRASUZ 1 developing xylem (xylem), E. globulus A2 developing xylem (gloA2) and E. globulus C3 xylem (gloC3). (TIFF 756 kb) [file 12864_2015_2322_MOESM1_ESM.tiff]

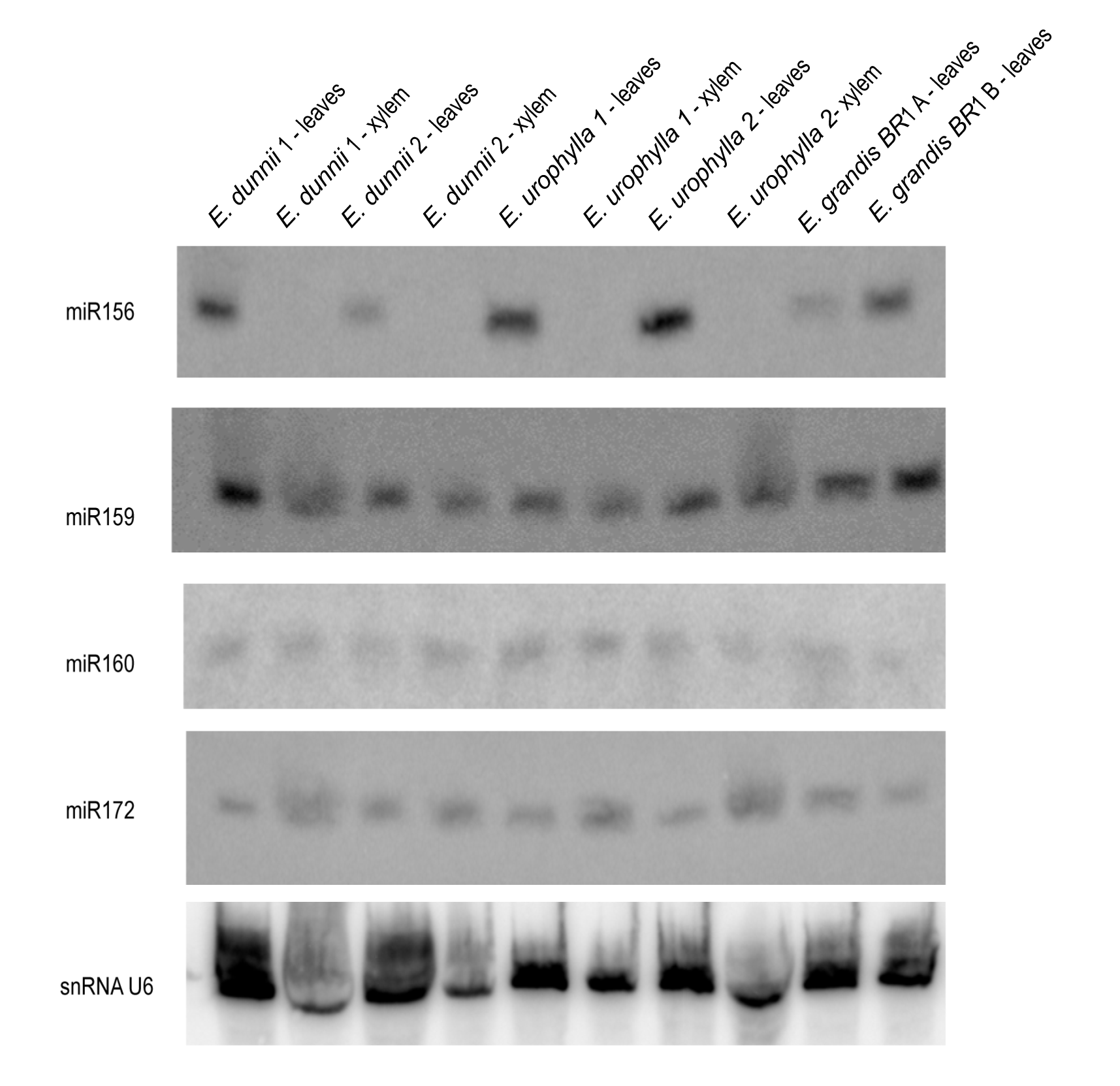

Supplement: Additional file 3: Figure S2. — Northern blot analysis of conserved micro RNAs. (TIF 4319 kb) [file 12864_2015_2322_MOESM3_ESM.tif]

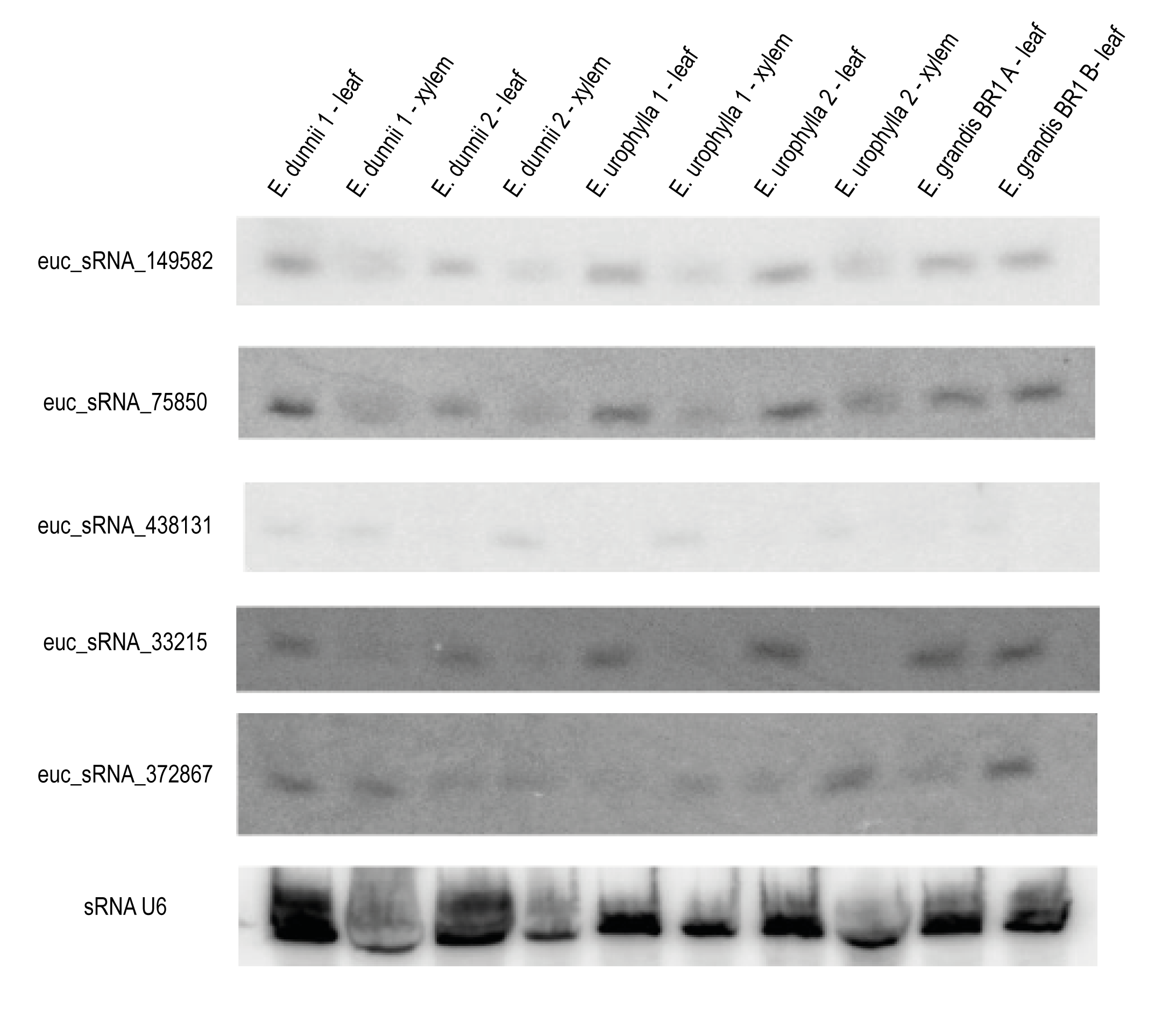

Supplement: Additional file 5: Figure S3. — Assessing small RNA conservation in Eucalyptus. (TIF 4142 kb) [file 12864_2015_2322_MOESM5_ESM.tif]
