# Supplementary material for: Genome-wide discovery and validation of Eucalyptus small RNAs reveals variable patterns of conservation and diversity across species of Myrtaceae
Source: BMC Genomics. 2015 Dec 29;16:1113. doi: 10.1186/s12864-015-2322-6 (PMC4696225; doi:10.1186/s12864-015-2322-6)
Supplement: Additional file 6: Table S3. — Eucalyptus miR159a targets predicted by psRNATarget. (DOCX 12 kb) [file 12864_2015_2322_MOESM6_ESM.docx]

Predicted targets for *Eucalyptus grandis* miR159a by psRNATarget. Transcript names (as described in Phytozome), E value for pair miRNA-target alignment, predicted mode of inhibition (cleavage of messenger RNA or translation inhibition) and PFAM protein domain code and name as retrieved from Biomart tool.

| ***Transcript name*** | ***E value*** | ***Inhibition*** | ***ID PFAM*** | ***Protein domain*** |
| --- | --- | --- | --- | --- |
| Eucgr.B03766.1 | 3.0 | cleavage | - | - |
| Eucgr.C02627.1 | 2.5 | translation | PF00931  PF00560  PF01582 | NB-ARC domain  LRR(leucine-rich repeat)  TIR domain |
| Eucgr.E01581.1 | 2.5 | cleavage | PF00249 | Myb-like DNA-binding domain |
| Eucgr.F01065.1 | 2.5 | cleavage | - | - |
| Eucgr.F01202.1 | 3.0 | cleavage | - | - |
| Eucgr.F03131.1 | 3.0 | Tradução | PF02990 | Endomembrane protein 70 |
| Eucgr.G03183.1 | 3.0 | cleavage | PF00249 | Myb-like DNA-binding domain |
| Eucgr.H04493.1 | 2.5 | cleavage | PF00501 | AMP-binding enzyme |
| Eucgr.J00789.1 | 2.5 | cleavage | PF02535 | ZIP Zinc transporter |
